# Effect of corneal cross-linking on biomechanical changes following transepithelial photorefractive keratectomy and femtosecond laser-assisted LASIK

**DOI:** 10.3389/fbioe.2024.1323612

**Published:** 2024-03-15

**Authors:** Wen Chen, FangJun Bao, Cynthia J. Roberts, Jia Zhang, Chong Wang, XueFei Li, JunJie Wang, Anas Ziad Masoud Abu Said, Kevin Nguelemo Mayopa, YaNi Chen, XiaoBo Zheng, Ashkan Eliasy, Ahmed Elsheikh, ShiHao Chen

**Affiliations:** ^1^ National Clinical Research Center for Ocular Diseases, Eye Hospital, Wenzhou Medical University, Wenzhou, China; ^2^ National Engineering Research Center of Ophthalmology and Optometry, Eye Hospital, Wenzhou Medical University, Wenzhou, China; ^3^ State Key Laboratory of Ophthalmology, Optometry and Vision Sicence, Eye Hospital, Wenzhou Medical University, Wenzhou, China; ^4^ The Institute of Ocular Biomechanics, WenZhou Medical University, Wenzhou, China; ^5^ Ophthalmology and Visual Sciences and Biomedical Engineering, The Ohio State University, Columbus, OH, United States; ^6^ School of Engineering, University of Liverpool, Liverpool, United Kingdom; ^7^ National Institute for Health Research (NIHR) Biomedical Research Centre for Ophthalmology, Moorfields Eye Hospital NHS Foundation Trust and UCL Institute of Ophthalmology, London, United Kingdom; ^8^ Beijing Advanced Innovation Center for Biomedical Engineering, Beihang University, Beijing, China

**Keywords:** corneal stiffness, tPRK, FS-LASIK, CXL, Corvis ST

## Abstract

**Purpose:** To evaluate the change in corneal biomechanics in patients with postoperative ectasia risk when combining two common laser vision correction procedures (tPRK and FS-LASIK) with cross-linking (in tPRK Xtra and FS-LASIK Xtra).

**Methods:** The study included 143 eyes of 143 myopic, astigmatic patients that were divided into non-cross-linked refractive surgery groups (non-Xtra groups, tPRK and FS-LASIK) and cross-linked groups (Xtra groups, tPRK Xtra and FS-LASIK Xtra) according to an ectasia risk scoring system. The eyes were subjected to measurements including the stress-strain index (SSI), the stiffness parameter at first applanation (SP-A1), the integrated inverse radius (IIR), the deformation amplitude at apex (DA), and the ratio of deformation amplitude between apex and 2 mm from apex (DARatio2mm). The measurements were taken preoperatively and at 1, 3, and 6 months postoperatively (pos1m, pos3m, and pos6m). Posterior demarcation line depth from the endothelium (PDLD) and from the ablation surface (DLA) were recorded at pos1m.

**Results:** SP-A1 significantly decreased, while IIR, deformation amplitude, and DARatio2mm increased significantly postoperatively in all four groups (*p* < 0.01)—all denoting stiffness decreases. In the FS-LASIK group, the changes in IIR, DA, and DARatio2mm were 32.7 ± 15.1%, 12.9 ± 7.1%, and 27.2 ± 12.0% respectively, which were significantly higher (*p* < 0.05) compared to 20.1 ± 12.8%, 6.4 ± 8.2%, and 19.7 ± 10.4% in the FS-LASIK Xtra group. In the tPRK group, the change in IIR was 27.3 ± 15.5%, significantly larger than 16.9 ± 13.4% in the tPRK Xtra group. The changes of SSI were minimal in the tPRK (−1.5 ± 21.7%, *p* = 1.000), tPRK Xtra (8.4 ± 17.9%, *p* = 0.053), and FS-LASIK Xtra (5.6 ± 12.7%, *p* = 0.634) groups, but was significant in the FS-LASIK group (−12.1 ± 7.9%, *p* < 0.01). After correcting for baseline biomechanical metrics, preoperative bIOP and the change in central corneal thickness (△CCT) from pre to pos6m, the changes in the IIR in both FS-LASIK and tPRK groups, as well as DA, DARatio2mm and SSI in the FS-LASIK group remained statistically greater than their corresponding Xtra groups (all *p* < 0.05). Most importantly, after correcting for these covariates, the changes in DARatio2mm in the FS-LASIK Xtra became statistically smaller than in the tPRK Xtra (*p* = 0.017).

**Conclusion:** The statistical analysis results indicate that tPRK Xtra and FS-LASIK Xtra effectively reduced the biomechanical losses caused by refractive surgery (tPRK and FS-LASIK). The decrease in corneal overall stiffness was greater in FS-LASIK than in tPRK, and the biomechanical enhancement of CXL was also higher following LASIK than after tPRK.

## 1 Introduction

Due to improvement in vision and high patient satisfaction, laser vision correction (LVC) surgeries have become increasingly popular in recent years. The loss of tissue due to ablation in surface treatments such as tPRK and the separation of a flap or cap in lamellar ablation procedures such as LASIK and SMILE, lead to reductions in corneal stiffness, which have led to some rare cases of corneal instability (Seiler et al., 1998; Malecaze et al., 2006; Randleman et al., 2006; Mattila and Holopainen, 2016). Since Seiler et al. (1998) reported the first iatrogenic keratectasia case after refractive surgery in 1998, which manifested as corneal progressive thinning and shape distortion, there have been more reports of ectasia with associated refractive error increases and loss of visual acuity. The incidence of ectasia after LASIK is between 0.04% and 0.60% (Xu et al., 2017), and much less after PRK (Roszkowska et al., 2017). SMILE, as a relatively new procedure, also has been reported a 0.15% incidence of ectasia post-surgery (Brar et al., 2021). Although rare, iatrogenic ectasia remains an extremely serious complication, which should be avoided.

Corneal cross-linking (CXL) is the most common method used to halt the progression of keratoconus and has been proven effective in stiffening the cornea in both *in vivo* and *ex vivo* testing (Spoerl et al., 1998; Sedaghat et al., 2018). In an attempt to improve the biomechanical integrity of the ablated cornea and reduce the incidence of iatrogenic keratectasia after refractive surgery, Kanellopoulos (2012) was the first to combine prophylactic high irradiance, short exposure CXL with LASIK. This was followed by reports of the clinical efficacy of combining prophylactic CXL with both PRK and SMILE, in procedures termed PRK Xtra and SMILE Xtra (Lee et al., 2017a; Torres-Netto et al., 2020).

Recognition of the importance of corneal biomechanics and the negative effects of LVC surgeries has led to attempts to quantify corneal stiffness *in vivo*, including the deformation parameters of the Corvis ST. These parameters included the deformation amplitude (DA), Stiffness Parameter (SP), and the Integrated Inverse Radius (IIR) (Vinciguerra et al., 2016; Roberts et al., 2017), all of which have been correlated with the cornea’s overall stiffness. A more recent development is the Corvis Stress-Strain Index (SSI), designed through finite element modeling to estimate the material stiffness of the cornea–rather than its overall stiffness–and seeks to characterize the non-linear stress-strain behavior and hence the tangent modulus at any intraocular pressure value (Eliasy et al., 2019). This latter point is of particular importance since corneal tissue is known to have non-linear pressure-deformation behavior and stress-strain behavior, and hence the tangent modulus (Et) does not maintain a constant value but increases gradually with load, stress, deformation, and strain (Qin et al., 2019).

In this study, we aimed to use Corvis ST biomechanical parameters, to evaluate corneal biomechanical response to two common LVC procedures, namely tPRK and FS-LASIK, and their variations that combine cross-linking with the tissue ablation procedures; tPRK Xtra and FS-LASIK Xtra.

## 2 Materials and methods

### 2.1 Study participants

**FIGURE 1:**
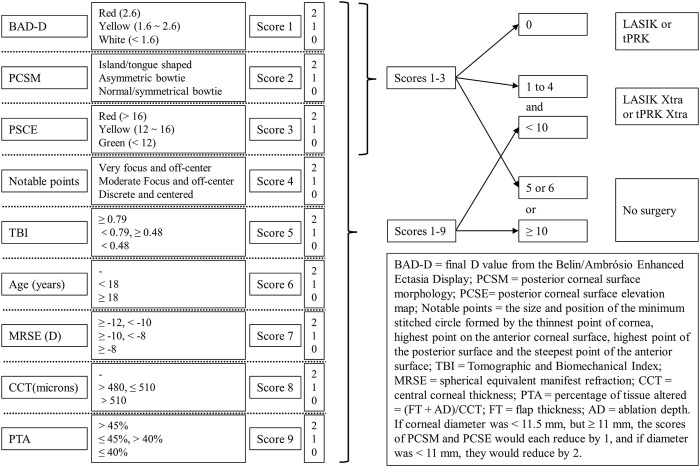
Chen ShiHao (CSH) scoring criteria used in preoperative assessment of patients.

The study followed the tenets of the Declaration of Helsinki and was approved by the Ethics Committee of the Eye Hospital, WMU. Only the right eye of each patient with no systemic or ocular condition, apart from the refractive error, was selected for analysis. A total of 177 patients who underwent corneal refractive surgery for myopia and astigmatism at the Eye Hospital of Wenzhou Medical University (WMU) were prospectively and consecutively enrolled in this study. Clinical examinations were conducted preoperatively(pre), and at 1 month(pos1m), 3 months(pos3m) and 6 months(pos6m) postoperative, follow-up data at these time points were available for 170, 160, and 143 patients, respectively. Only the 143 patients with complete records were included in the follow-up analysis. Among these patients, 37 received transepithelial PRK (tPRK), 35 underwent tPRK Xtra (tPRK combined with CXL), 35 underwent FS-LASIK, and 36 underwent FS-LASIK Xtra (FS-LASIK combined with CXL). In the Xtra procedures, tPRK and FS-LASIK were combined with accelerated CXL based on the criteria listed in Figure 1, which combined the surgeon’s (CSH) personal experience with an ectasia risk scoring system (Randleman et al., 2008; Chan et al., 2018). These patient inclusion criteria were roughly the same as described previously (Zhang et al., 2022), but with slight modifications. The figure shows the criteria used in determining whether to use CXL with FS-LASIK or tPRK, and the cases in which surgery would not be recommended. In other words, patients with eyes at risk of developing ectasia after LVC underwent either FS-LASIK Xtra or tPRK Xtra to improve the mechanical stability of the cornea postoperatively, while patients without postoperative ectasia risk underwent either FS-LASIK or tPRK. The choice between tPRK and FS-LASIK, or between tPRK Xtra and FS-LASIK Xtra was based on the clinical judgment of the surgeon. Patients’ informed and signed consent was received after explaining the advantages and disadvantages of the study. All surgeries were performed by the same experienced surgeon (CSH).

### 2.2 Surgical techniques

In the tPRK group, ablation of corneal epithelium (ablated depth was set at 50 μm) and stroma was performed in a single step under the aberration-free mode of an Amaris 750 Hz excimer laser (Schwind eye-tech-solutions, Kleinostheim, Germany). In the FS-LASIK procedure, the lamellar flap was separated using FEMTO LDV Crystal Line femtosecond laser (Ziemer Ophthalmic Systems AG, Port, Switzerland). The flaps had a superior hinge, and their thickness ranged from 90 to 110 µm and diameter from 8.5 to 9.0 mm. The remaining specific parameters utilized in both procedures were maintained at a constant level, encompassing a stromal ablation rate of 12 mm/s, an edge-cutting speed of 6 mm/s, a repetition rate of 10 MHz, a pulse duration of 250 fs, and a spot energy of 880 mW. The ablation was then performed using the Amaris 750 Hz excimer laser. Residual stromal bed thickness (RSB) was recorded from surgery planning/treatment printouts. In calculating the RSB, the flap thickness was excluded in the two FS-LASIK groups while the epithelium thickness was not included in the two tPRK groups.

In the Xtra procedures, laser ablation was completed first before treating the residual corneal stroma with isotonic 0.22% riboflavin solution (VibeX Xtra; Avedro, Waltham, MA) for 100–120 s. Excess riboflavin was rinsed off with a saline solution before exposure to UVA in a continuous irradiation protocol at a power of 30 mW/cm^2^ and a total dose of 2.1–2.7 J/cm^2^. As shown in Table 1, various soaking times of riboflavin and total doses used in CXL procedure were dependent on the sum of the scores in Figure 1. After UVA irradiation, the corneal flap was re-placed to cover the residual corneal stroma in the FS-LASIK Xtra group. This procedure was different from that suggested by Avedro, the CXL machine manufacturer, in which UVA irradiation was to be applied after re-placing the flap on the residual stroma. This change was implemented to ensure that the full stiffening effect of CXL is realized in the residual stroma and not partly consumed in the flap, which ceases to contribute mechanically to the stroma once separated (Kanellopoulos et al., 2015; Randleman et al., 2017). This form of FS-LASIK Xtra was approved by Wenzhou Eye Hospital before using it in practice.

**TABLE 1:** CXL settings variations with different ectasia risk scores.

Cumulative risk score	Risk classification	Riboflavin soak time(s)	Total dose (J/cm^2^)
1 to 3	1	100	2.1
4 to 6	2	110	2.4
7 to 9	3	120	2.7

### 2.3 Data collection

The examinations included measurements by the Corvis ST non-contact tonometer (CVS, Oculus Optikgeräte GmbH, Wetzlar, Germany). Surgical parameters including the optical zone diameter (OZD), manifest refractive error correction (REC) and best-corrected visual acuity (BCVA) were also recorded from surgery planning/treatment printouts. REC was converted into spherical equivalent (SE). The safety index of both procedures, defined as the quotient of the postoperative BCVA divided by the preoperative BCVA, denoted a procedure as safe with values equal to or greater than one. Corneal haze was examined in follow-up by a BQ900 slit lamp (Haag-Streit, Germany), and assessed using the following scoring criteria: 0, normal cornea; 0.5, slight corneal haze; 1, mild haze; 2, moderate opacity or scarring; 3, severe corneal opacity, but clear iris visibility; 4, opaque cornea and corneal ulcer; 5, corneal rupture and necrotizing stromal keratitis (Kim et al., 2006). Central corneal thickness (CCT) and corneal densitometry was measured with a Pentacam (Oculus Optikgerate GmbH). For the two Xtra groups, optical coherence tomography (OCT) (SD-OCT; RTVue-XR; Optovue, Inc., Fremont, CA) scanning was performed to locate the demarcation line within the stroma at 1, 3, 6 months post-surgery. The demarcation line was then used to determine the central thickness of the uncross-linked tissue that included the posterior part of the central stroma and the whole endothelium. This thickness is henceforth called the *posterior* demarcation line depth, or PDLD. This depth was used instead of the commonly used demarcation line depth (DLD), which focuses on the *anterior* part of the corneal thickness in order to avoid the possible confusion that can be created by the flap which had not been cross-linked and would not in any case contribute to the corneal biomechanical behavior. The demarcation line depth from the ablation surface (DLA) was then calculated as RSB—PDLD. The endothelial cell count (ECC) was also obtained using specular microscopy (SP-3000P, Topcon, Tokyo, Japan) at 6 months post-operation. Patients who were unwilling to participate or did not complete the 6 months postoperative follow-up were not included in the study. Ablated stromal depth (ASD) was recorded from surgery planning/treatment printouts. For tPRK, ASD was defined as ablation depth subtracted by central ablated epithelium thickness.

### 2.4 Postoperative care

**FIGURE 2:**
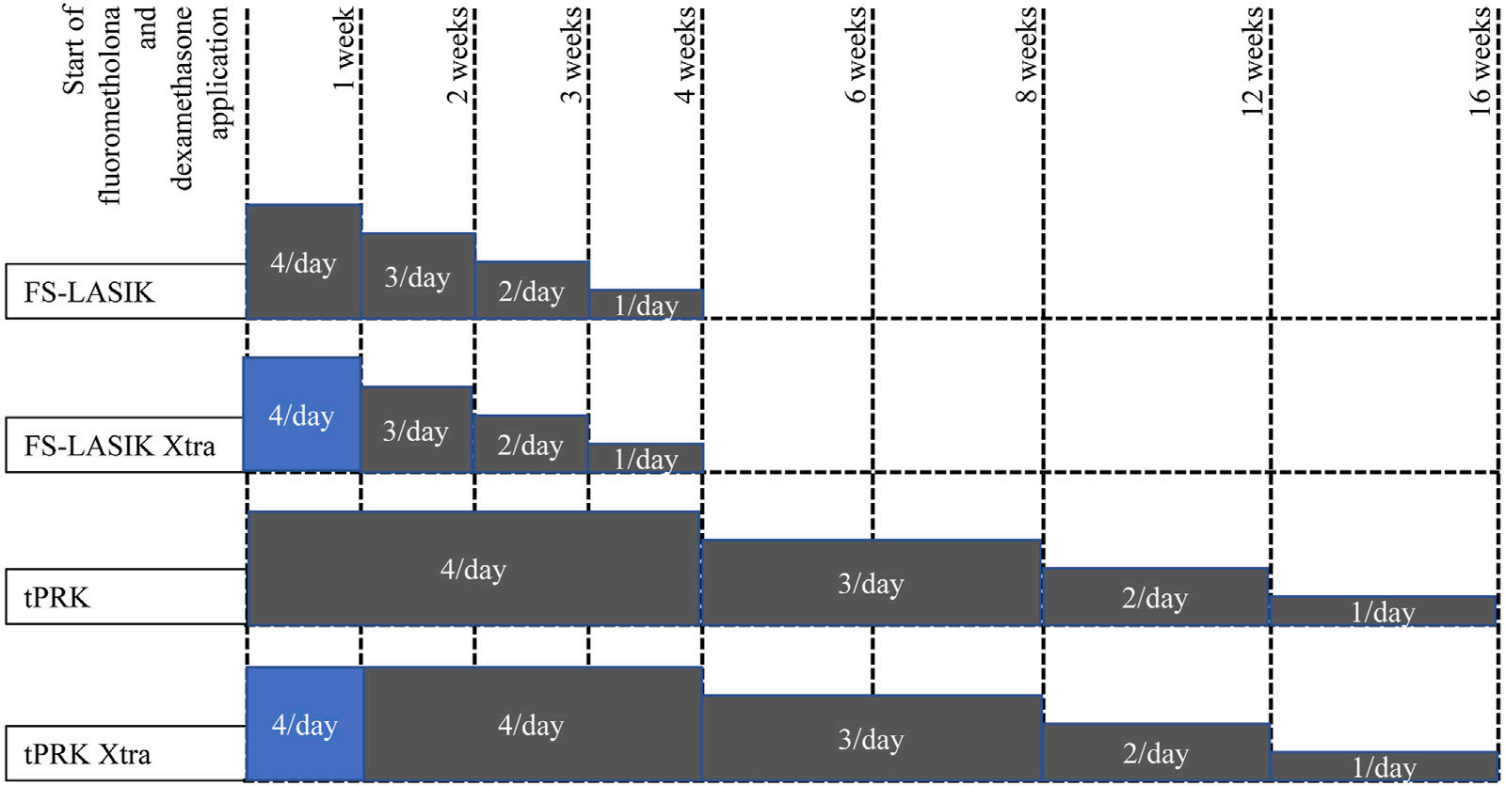
Tapering of fluorometholone and dexamethasone application following the four surgeries, grey color means fluorometholone usage while blue color means dexamethasone usage.

The postoperative care was similar for the four procedures. One drop of tobramycin/dexamethasone (Tobradex; Alcon, TX, United States) was instilled at the surgical site. A bandage contact lens (Acuvue Oasys; Johnson & Johnson, FL, United States) was then placed on the cornea and kept for 1 day in the FS-LASIK and FS-LASIK Xtra groups until complete re-epithelization in the tPRK and tPRK Xtra groups–typically between 5 and 7 days. Topical levofloxacin 0.5% (Cravit; Santen, Osaka, Japan) was used until the bandage lens was taken off. This was followed by application of fluorometholone 0.1% (Flumetholon; Santen, Osaka, Japan), topical levofloxacin 0.5% (Cravit; Santen, Osaka, Japan), and dexamethasone (Tobradex; Alcon, Rijksweg, Belgium) whose frequency, duration, and tapering regime varied between the procedures as shown in Figure 2.

### 2.5 Biomechanical evaluation

All Corvis ST exams were taken three times in a sitting position with undilated pupils by two experienced examiners (WH and HNL) in the same half-day session to minimize diurnal effects. Five biomechanical parameters were chosen for analysis including the SSI, the deformation amplitude at the apex (DA), the ratio of deformation amplitude between the apex and 2 mm from the apex (DARatio2mm). The fourth parameter was the integrated inverse radius (IIR), which represents the integrated sum of inverse concave radius between the first and the second applanation events. The parameters also included the stiffness parameter at first applanation (SP-A1) calculated as the difference between the adjusted air puff pressure at the first applanation (Adj AP1) and the biomechanically corrected intraocular pressure (bIOP) divided by the defection amplitude at the first applanation (A1 DeflAmp) (Eq. 1, Roberts et al., 2017).

$$\text{SP}-A1=\left(\text{adjAP}1\;–\text{ bIOP}\right)/\left(A1\text{DeflAmp}\right)$$ (1)

While SSI was developed as a measure of the cornea’s material stiffness using finite element analysis, the other four parameters were known to be correlated with the tissue’s overall stiffness (Eliasy et al., 2019; Esporcatte et al., 2020) Furthermore, while increases in SSI and SP-A1 indicated stiffness increases, increases in IIR, DA and DARatio2mm pointed at stiffness reductions (Esporcatte et al., 2020).

### 2.6 Statistical analysis

All analyses were performed using the PASW Statistics 20.0 (SPSS Inc., Chicago, United States). Comparisons by post-op time within each of the four surgery methods were made using the MANOVA of repeated measurements. Comparisons of the biomechanical differences between pre-and post-surgery were carried out using two-way analysis of variance (ANOVA) and, two-way analysis of covariance (ANCOVA) with baseline biomechanical metrics (the biomechanical parameters recorded at the pre-surgery stage), preoperative bIOP and the change in central corneal thickness (△CCT) from pre to each follow-up period (pos1m, pos3m, and pos6m) as covariates. If the data did not fulfill the necessary assumptions of ANCOVA, GLM was used. Analysis of multiple groups was done with one-way ANOVA when the biomechanical comparisons involved the non-Xtra group and non-corresponding Xtra group (e.g., tPRK vs. FS-LASIK Xtra). The frequencies of the categorical variable gender were arranged in a 3 × 2 contingency table and the Chi-square test of independence was used to compare them. A *p*-value of less than 0.05 was considered statistically significant.

## 3 Results

The four groups (tPRK, tPRK Xtra, FS-LASIK, and FS-LASIK Xtra) were matched (all *p* > 0.05) in age, gender ratio, CCT, OZD, SE, ASD, and bIOP (Table 2). Further, the tPRK group and the tPRK Xtra group were matched in RSB thickness, while the FS-LASIK group and the FS-LASIK Xtra group were matched in both RSB and flap thickness (all *p* > 0.05). The risk classifications for the tPRK Xtra and FS-LASIK Xtra groups were not significantly different (*p* = 0.123). Table 3 shows the different baselines in Xtra and their corresponding non-Xtra groups, the table also shows the changes in corneal response parameters recorded before and after the four surgeries.

**TABLE 2:** Basic biometric parameters of the four surgery groups.

Parameters	tPRK	tPRK Xtra	FS-LASIK	FS-LASIK Xtra	F	P
Age(years)	25.1 ± 4.1	23.1 ± 5.4	25.3 ± 4.3	24.3 ± 6.4	1.348	0.261
gender ratio	11/26	15/20	18/17	10/26	5.711	0.127
CCT(μm)	542.8 ± 32.0	535.9 ± 33.1	549.3 ± 28.0	546.7 ± 27.4	1.302	0.276
OZD(mm)	6.36 ± 0.39	6.16 ± 0.48	6.39 ± 0.29	6.28 ± 0.45	2.265	0.084
SE(D)	-5.31 ± 1.96	-5.44 ± 2.02	-5.46 ± 1.70	-5.13 ± 1.66	0.231	0.874
ASD(μm)	85.6 ± 22.0	84.2 ± 19.5	82.1 ± 23.1	77.8 ± 18.5	0.957	0.415
bIOP(mmHg)	15.0 ± 1.8	15.4 ± 2.0	14.7 ± 1.8	15.6 ± 2.2	1.462	0.228
Risk classification	-	1.51 ± 0.70	-	1.36 ± 0.59	2.456	0.123

Gender ratio was calculated as Male/Female, CCT, means central corneal thickness; OZD, means optical zone diameter; SE, means spherical equivalent of manifest refractive error correction; ASD, means Ablated stromal depth (ASD), bIOP, means biomechanically corrected intraocular pressure provided by Corvis ST.

**TABLE 3:** Change in corneal biomechanical metrics and CCT after different surgeries of the four surgery groups.

	Stages	tPRK	tPRK Xtra	FS-LASIK	FS-LASIK Xtra	tPRK VS. tPRK Xtra	tPRK VS. LASIK	tPRK Xtra VS. LASIK Xtra	LASIK VS. LASIK Xtra
		Means ± SD				*p*-Value			
SP-A1(mmHg/mm)	Pre	105.97 ± 16.83	107.84 ± 18.6	104.61 ± 14.85	114.1 ± 19.02	1.00	1.00	0.86	0.43
	Pos1m	75.69 ± 13.88	83.78 ± 25.97	76.22 ± 17.84	81.25 ± 15.68	0.42	1.00	1.00	1.00
	Pos3m	77.05 ± 16.70	79.07 ± 16.06	74.66 ± 15.78	83.09 ± 11.62	1.00	1.00	1.00	0.12
	Pos6m	77.75 ± 19.25	80.08 ± 17.50	72.31 ± 16.13	83.27 ± 14.74	1.00	1.00	1.00	0.05*
IIR(mm^−1^)	Pre	8.65 ± 1.21	9.32 ± 0.94	8.25 ± 0.92	9.09 ± 0.94	0.04*	0.38	1.00	<0.01**
	Pos1m	10.52 ± 1.24	10.72 ± 1.27	10.68 ± 0.74	11.22 ± 0.92	1.00	1.00	0.38	0.26
	Pos3m	10.73 ± 1.32	11.1 ± 1.21	10.81 ± 0.97	10.96 ± 1.09	1.00	1.00	1.00	1.00
	Pos6m	10.89 ± 1.13	10.83 ± 1.1	10.84 ± 0.91	10.85 ± 0.82	1.00	1.00	1.00	1.00
DA (mm)	Pre	1.03 ± 0.08	1.07 ± 0.10	1.02 ± 0.07	1.05 ± 0.10	0.34	1.00	1.00	0.42
	Pos1m	1.08 ± 0.08	1.08 ± 0.15	1.10 ± 0.07	1.14 ± 0.09	1.00	1.00	0.16	0.44
	Pos3m	1.10 ± 0.09	1.14 ± 0.14	1.13 ± 0.06	1.12 ± 0.07	0.53	1.00	1.00	1.00
	Pos6m	1.14 ± 0.09	1.14 ± 0.11	1.15 ± 0.07	1.11 ± 0.08	1.00	1.00	0.65	0.51
DARatio2mm	Pre	4.62 ± 0.47	4.54 ± 0.46	4.56 ± 0.44	4.45 ± 0.49	1.00	1.00	1.00	1.00
	Pos1m	5.57 ± 0.60	5.02 ± 0.61	5.87 ± 0.55	5.41 ± 0.53	0.00**	0.17	0.03*	0.01**
	Pos3m	5.42 ± 0.56	5.21 ± 0.45	5.79 ± 0.58	5.30 ± 0.51	0.49	0.01**	1.00	<0.01**
	Pos6m	5.40 ± 0.58	5.20 ± 0.63	5.78 ± 0.52	5.30 ± 0.55	0.86	0.04*	1.00	0.01**
SSI	Pre	0.95 ± 0.14	0.87 ± 0.12	0.98 ± 0.13	0.87 ± 0.12	0.03*	1.00	1.00	0.01**
	Pos1m	0.94 ± 0.13	0.93 ± 0.19	0.91 ± 0.10	0.88 ± 0.13	1.00	1.00	0.13	1.00
	Pos3m	0.93 ± 0.15	0.91 ± 0.20	0.86 ± 0.11	0.91 ± 0.13	0.34	0.02*	1.00	1.00
	Pos6m	0.93 ± 0.20	0.94 ± 0.19	0.85 ± 0.09	0.91 ± 0.12	1.00	0.27	1.00	0.54
CCT (μm)	Pre	554.84 ± 33.62	545.97 ± 34.92	559.52 ± 28.61	555.73 ± 26.54	1.00	1.00	0.43	1.00
	Pos1m	451.70 ± 44.02	437.15 ± 43.94	459.88 ± 35.18	466.64 ± 24.46	0.63	1.00	0.01**	1.00
	Pos3m	450.61 ± 45.43	445.40 ± 44.38	464.60 ± 34.09	469.32 ± 24.98	1.00	0.74	0.06	1.00
	Pos6m	448.08 ± 42.23	450.38 ± 44.35	463.04 ± 35.10	470.65 ± 23.47	1.00	0.55	0.12	1.00

SP-A1, means the stiffness parameter at first applanation; IIR, means integrated inverse radius; DA means the deformation amplitude at the apex, DARatio2mm means the ratio of deformation amplitude between the apex and 2 mm from the apex, SSI, means the stress-strain index; CCT, means central corneal thickness; bIOP, means biomechanically corrected intraocular pressure provided by Corvis ST, * Means *p* < 0.05, ** means *p* < 0.01.

**FIGURE 3:**
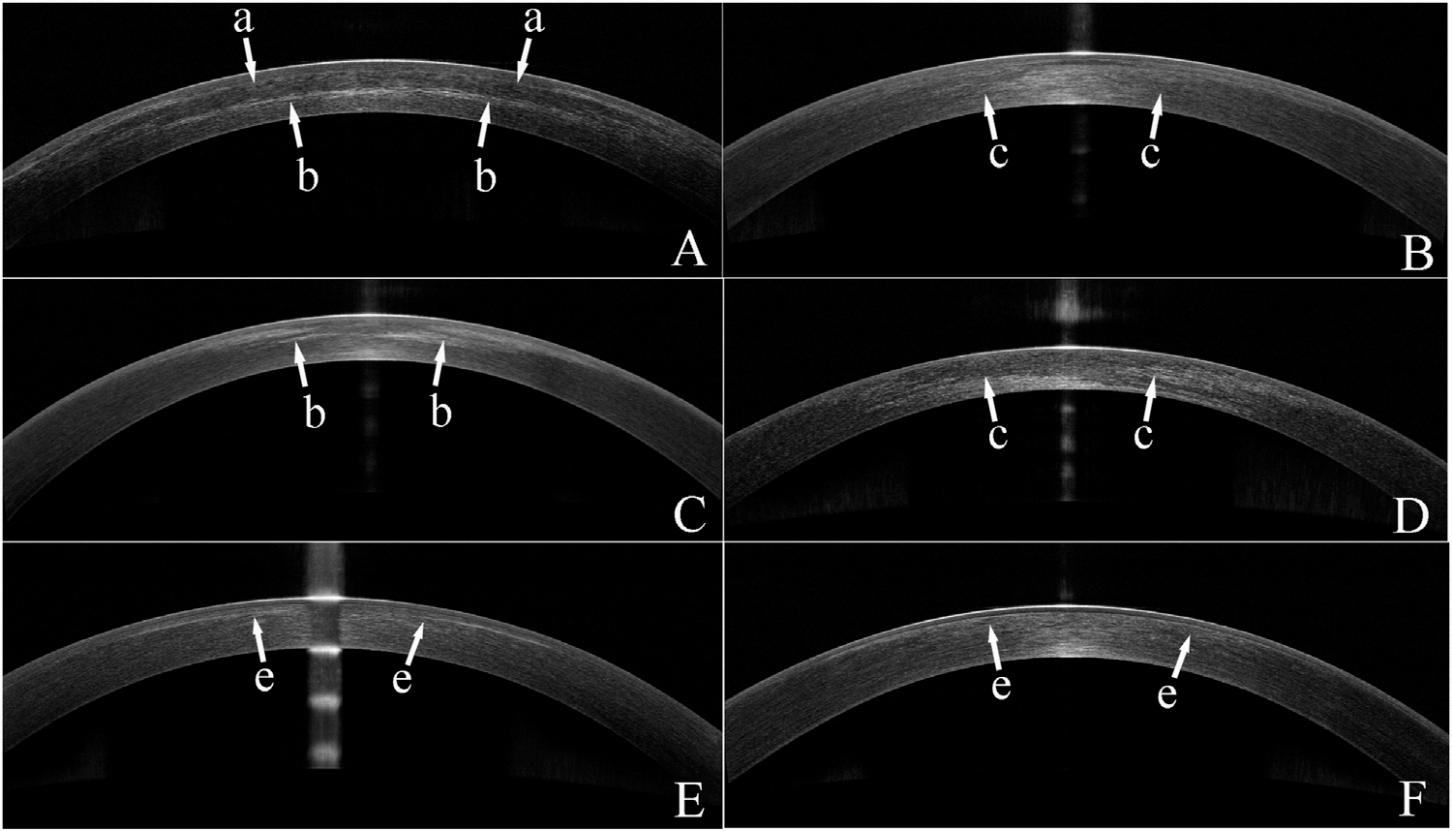
Optical coherence tomography (OCT) images of eyes from the two Xtra groups. **(A)** An OCT image of a FS-LASIK Xtra eye with a clear demarcation line at 1 month post surgery(pos1m). The arrows with an “a” indicate the interface between the flap and the residual stroma. Arrows with a “b” indicate the demarcation line. **(B)** An OCT image of a FS-LASIK Xtra eye with a faint demarcation line identified by “c” arrows at pos1m. **(C)** An OCT image of a tPRK Xtra eye showing a clear demarcation line indicated by arrows with a “b” at pos1m. **(D)** An OCT image of a tPRK Xtra eye with a faint demarcation line indicated by “c” arrows at pos1m. **(E)**. An OCT image of a FS-LASIK Xtra eye, recorded at 3 months post-surgery. Arrows with a “e” indicate the haze at the interface. **(F)**. An OCT image of a tPRK Xtra eye, recorded at 6 months post-surgery. Arrows with a “e” indicate the haze in the stroma.

None of patients developed serious complications, and no difference was found in spherical equivalent (SE) among all groups at pos6m. The safety index at pos6m was 1.12 ± 0.11 in tPRK, 1.01 ± 0.06 in tPRK Xtra, 1.06 ± 0.12 in FS-LASIK and 1.03 ± 0.08 in FS-LASIK Xtra. At pos1m, 18.9% (7/37) of eyes in the tPRK group, 65.7% (23/35) in the tPRK Xtra group, and 31.4% (11/35) in the FS-LASIK Xtra group exhibited grade 0.5 haze. Additionally, 20.0% (7/35) of eyes in the tPRK Xtra had grade 1 haze. By the pos3m, haze had persisted only in the Xtra groups, with 60.0% (21/35) of tPRK Xtra eyes and 45.7% (16/35) in FS-LASIK Xtra eyes showing grade 0.5 haze, and 2.9% (1/35) in the FS-LASIK Xtra with grade 1 haze. At pos6m, 25.7% (9/35) of eyes in the tPRK Xtra and 14.3% (5/35) in the FS-LASIK Xtra still had grade 0.5 haze. The residual stromal bed thickness pre-CXL in FS-LASIK Xtra group was significantly lower than in tPRK Xtra group (*p* < 0.001, 374.3 ± 25.7 μm vs. 401.7 ± 40.1 μm). The appearance of a stromal demarcation line was observed in all Xtra eyes in the OCT scans recorded at pos1m (Figure 3). The mean central stromal PDLD was 232.4 ± 50.3 μm (range 128–314 μm), and 269.8 ± 64.6 μm (range 169–393 μm) in FS-LASIK Xtra and tPRK Xtra group, respectively. The differences in PDLD between the two groups were significant (*p* = 0.008). DLA in FS-LASIK Xtra was not different from the tPRK Xtra group (*p* = 0.543, 142.3 ± 52.5 μm vs. 134.9 ± 48.2 μm). In the FS-LASIK Xtra group, the demarcation line was well defined in 86.1% (31/36) of the eyes (Figure 3A), while 13.9% (5/36) had a faint line (Figure 3B). The corresponding ratios in the tPRK Xtra group were 57.1% (20/36) and 42.9% (15/36) (Figures 3C, D). The faint demarcation lines were still observable in 77.1% (27/35) and 60.0% (21/35) in the tPRK Xtra group, at pos3m and pos6m, respectively. In comparison, the FS-LASIK Xtra group demonstrated a lower observable incidence, with 61.1% (22/36) of eyes at pos3m and only 11.1% (4/36) at pos6m exhibiting faint demarcation lines. The ECC in FS-LASIK Xtra remained similar (*p* > 0.05) before (2,857 ± 344 cells/mm^2^) and after (2,742 ± 296 cells/mm^2^) surgery, and the same finding was true in the tPRK Xtra group (2,786 ± 225 cells/mm^2^ vs. 2,790 ± 264 cells/mm^2^). At pos1m, the tPRK Xtra group exhibited a significant increase in mean corneal densitometry over the total area compared to preoperative values (*p* = 0.015). In contrast, corneal densitometry remained stable among the tPRK, FS-LASIK, and FS-LASIK Xtra (*p* = 0.674, 0.391 and 1.000, respectively). In contrast, at pos6m and compared to preoperative values, the mean corneal densitometry over the total area in both the tPRK Xtra and FS-LASIK decreased significantly (*p* < 0.001 and 0.026, respectively), while there were no significant differences in the tPRK (*p* = 0.218) and FS-LASIK Xtra (*p* = 1.000). The specific means, standard deviations and *p* values can be found in Supplementary Tables S3, S4.

**FIGURE 4:**
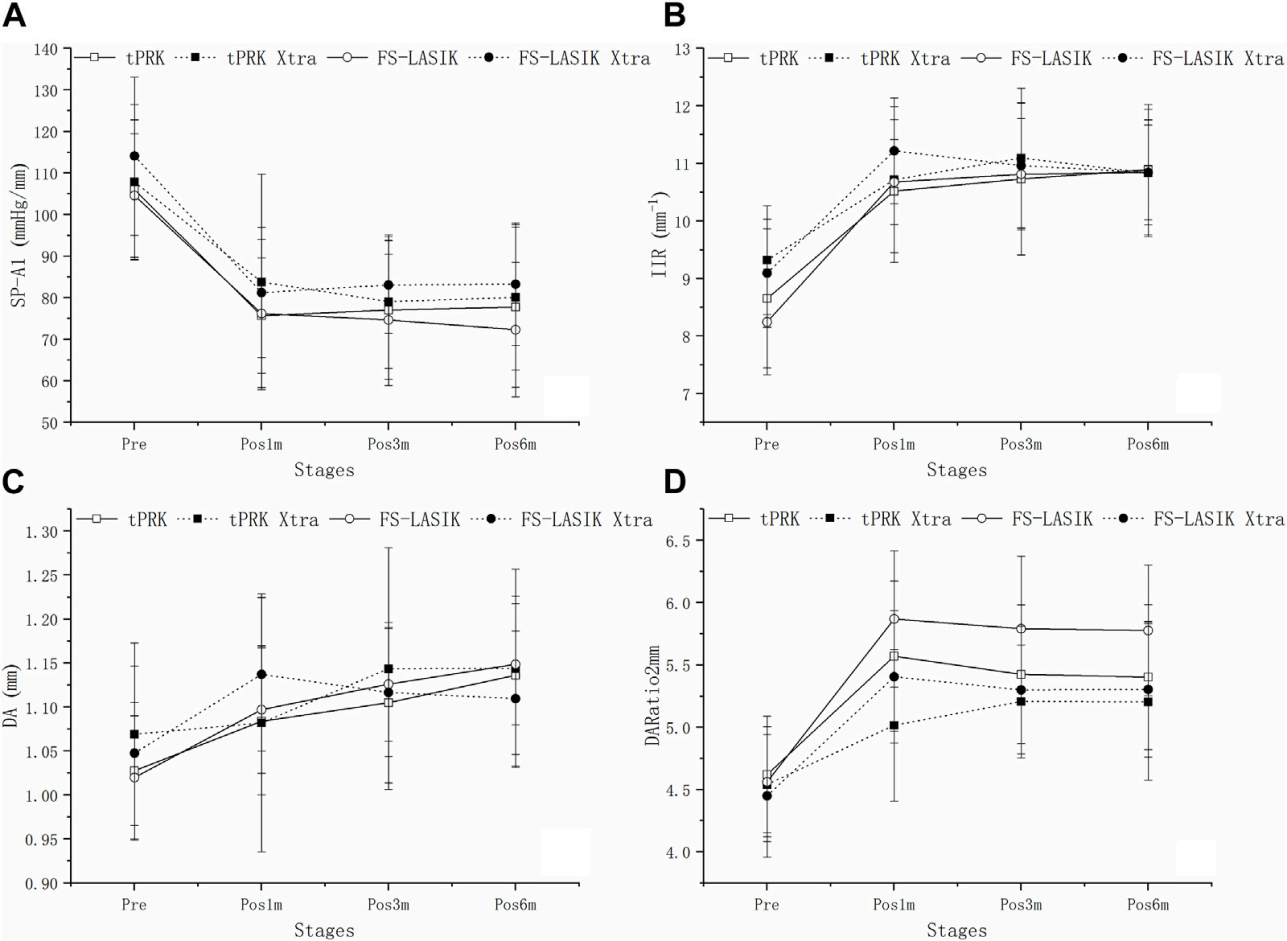
Change of SP-A1, IIR, DA and DARatio2mm throughout all follow up stages in the four groups. **(A)**: Change of SP-A1 throughout all follow up stages in the four groups. **(B)**: Change of IIR throughout all follow up stages in the four groups. **(C)**: Change of DA throughout all follow up stages in the four groups. **(D)**: Change of DARatio2mm throughout all follow up stages in the four groups.

Figure 4A shows a decrease in SP-A1 at pos1m compared with the pre-surgery stage (pre vs. pos1m, all *p* < 0.01). SP-A1 then remained stable (pos1m vs. pos3m: tPRK, *p* = 1.000; tPRK Xtra, *p* = 0.515; FS-LASIK, *p* = 1.000; FS-LASIK Xtra, *p* = 1.000. pos3m vs. pos6m: tPRK, *p* = 1.000; tPRK Xtra, *p* = 1.000; FS-LASIK, *p* = 1.000; FS-LASIK Xtra, *p* = 1.000. pos1m vs. pos6m: tPRK, *p* = 1.000; tPRK Xtra, *p* = 1.000; FS-LASIK, *p* = 0.737; FS-LASIK Xtra, *p* = 1.000) throughout follow up in all four groups. The specific means ± standard deviations and *p* values can be found in Table 3; Supplementary Table S2, respectively. Further, Table 4 shows a comparison of the difference in corneal biomechanical metrics between pre and pos6m (Δ, pos6m-pre) for the four surgery groups. There was no significant difference between the four groups in ΔSP-A1 between pre and pos6m (F = 0.911, *p* = 0.438). After correction for △CCT from pre to pos6m, preoperative bIOP and baseline SP-A1, ΔSP-A1 between pre and pos6m in FS-LASIK group became statistically higher than in the tPRK group (*p* = 0.005, Table 4), meaning greater reduction in SP-A1 stiffness in FS-LASIK than in tPRK.

**TABLE 4:** Comparison (*p*-value) of the difference (Δ) of corneal biomechanical metrics between pre and pos6m between the four surgery groups.

	Before baseline correction				After baseline correction			
	tPRK vs. FS-LASIK	tPRK Xtra vs. FS-LASIK Xtra	tPRK vs. tPRK Xtra	FS-LASIK vs. FS-LASIK Xtra	tPRK vs. FS-LASIK	tPRK Xtra vs. FS-LASIK Xtra	tPRK vs. tPRK Xtra	FS-LASIK vs. FS-LASIK Xtra
ΔSP-A1(mmHg/mm)	0.075	0.349	0.888	0.327	0.005**	0.185	0.768	0.235
ΔIIR (mm)	0.095	0.335	0.004**	<0.001**	0.089	0.327	0.003**	<0.001**
ΔDA (mm)	0.106	0.483	0.069	<0.001**	0.098	0.476	0.063	<0.001**
ΔDARatio2mm	<0.001**	0.092	0.288	0.001**	<0.001**	0.017*	0.531	0.002**
ΔSSI	0.008**	0.455	0.009**	<0.001**	0.008**	0.323	0.236	0.007**

Baseline correction means correcting for baseline biomechanical parameter, preoperative bIOP, and the change in central corneal thickness (△CCT) from pre to pos6m of in each group, SP-A1, means the stiffness parameter at first applanation; IIR, means integrated inverse radius, DARatio2mm means the ratio of deformation amplitude between the apex and 2 mm from the apex, SSI, means the stress-strain index, * Means *p* < 0.05, ** means *p* < 0.01.

**FIGURE 5:**
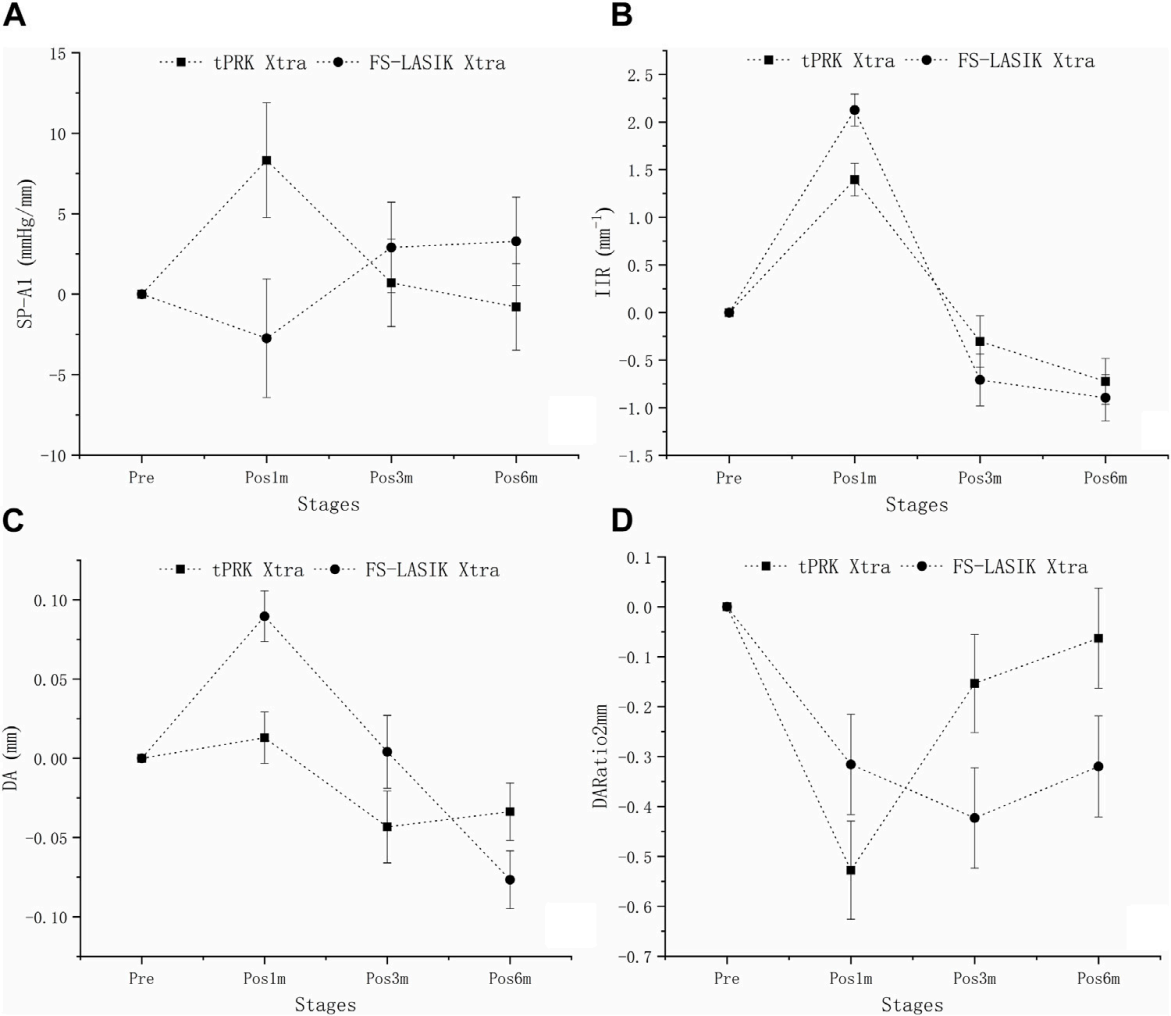
Isolating the effects of CXL: the estimated changes in SP-A1, IIR, DA, and DARatio2mm at all follow-up stages across the four groups. These changes are adjusted for preoperative bIOP, baseline biomechanical metrics, and the change in central corneal thickness (△CCT) from baseline to each follow-up stage. **(A)**: Isolating the effects of CXL: the estimated changes in SP-A1 at all follow-up stages across the four groups. **(B)**: Isolating the effects of CXL: the estimated changes in IIR at all follow-up stages across the four groups. **(C)**: Isolating the effects of CXL: the estimated changes in DA at all follow-up stages across the four groups. **(D)**: Isolating the effects of CXL: the estimated changes in DARatio2mm at all follow-up stages across the four groups.

Figure 4B shows that IIR exhibited significant increases (denoting stiffness reductions) from pre to pos1m in all surgery groups (all *p* < 0.01). This was followed by significant post-op changes in both the tPRK (pos1m vs. pos6m, *p* = 0.033) and tPRK Xtra (pos1m vs. pos3m, *p* = 0.037) groups, Figure 4B. The specific means ± standard deviations and *p* values can be found in Table 3; Supplementary Table S2, respectively. In contrast, IIR remained stable in the FS-LASIK and FS-LASIK Xtra groups (all *p* > 0.05, Figure 4B). The change in IIR between pre and pos6m (∆IIR) was smallest in tPRK Xtra (1.51 ± 1.09 mm^−1^, or 16.9 ± 13.4%), which was comparable to FS-LASIK Xtra (1.75 ± 0.95 mm^−1^, or 20.1 ± 12.8%, *p* = 1.000), Figure 4B; Table 4. ∆IIR in tPRK Xtra between pre and pos6m was also significantly lower in tPRK (2.24 ± 1.07 mm^−1^, or 27.3 ± 15.5%, *p* = 0.021) than in FS-LASIK (2.60 ± 0.97 mm^−1^, or 32.7 ± 15.1%, *p* < 0.001). The stiffening effect of CXL on IIR was statistically significant in tPRK (tPRK vs. tPRK Xtra, *p* = 0.004) and in FS-LASIK (FS-LASIK vs. FS-LASIK Xtra, *p* < 0.001). Similarly, after correction for △CCT from pre to pos6m, preoperative bIOP and baseline IIR, the stiffening effect of CXL on FS-LASIK (∆IIR: FS-LASIK vs. FS-LASIK Xtra, *p* < 0.001) and tPRK (∆IIR: tPRK vs. tPRK Xtra, *p* = 0.003) remained significant, Table 4. This highlights the distinct stiffening effects of corneal cross-linking in different LVC procedures, Figure 5B.

Figure 4C shows that DA increased at pos1m compared with the pre-surgery stage, indicating stiffness reductions in the presence of a stable IOP, in all groups (*p* < 0.01) except tPRK Xtra (*p* = 1.000). DA then continued to increase post-surgery in tPRK (pos1m vs. pos6m, *p* = 0.005), tPRK Xtra (pos1m vs. pos6m, *p* = 0.001) and FS-LASIK (pos1m vs. pos6m, *p* = 0.005), but was stable (*p* > 0.05) in the FS-LASIK Xtra group, Figure 4C; Table 3; Supplementary Table S2. Further, as shown in Figure 4C; Table 4, the change in DA between pre and pos6m (ΔDA) was smallest in FS-LASIK Xtra (0.06 ± 0.08 mm, or 6.4 ± 8.2%), similar to tPRK Xtra (0.07 ± 0.09 mm, or 7.3 ± 8.2%, *p* = 1.000) and tPRK (0.11 ± 0.08 mm, by 10.8 ± 7.6%, *p* = 0.069), while it was significantly higher in FS-LASIK (0.13 ± 0.07 mm, by 12.9 ± 7.1%, *p* < 0.001). Similarly, after correction for △CCT from pre to pos6m, preoperative bIOP and baseline DA, the stiffening effect of CXL on DA remained significant in the FS-LASIK group (FS-LASIK vs. FS-LASIK Xtra, *p* < 0.001), and not in tPRK (tPRK vs. tPRK Xtra, *p* = 0.063), Table 4. The results suggest that CXL may be more effective in enhancing the stiffness in FS-LASIK corneas compared with tPRK, Figure 5C.

There were also significant increases in DARatio2mm between pre and pos1m, indicating stiffness reductions, in all groups (all *p* < 0.01), and this trend remained valid over the rest of the follow-up period, Figure 4D. The specific means ± standard deviations and *p* values can be found in Table 3; Supplementary Table S2, respectively. Further, the increases in DARatio2mm between pre and pos6m (ΔDARatio2mm) were highest in FS-LASIK (1.22 ± 0.51), followed by FS-LASIK Xtra (0.85 ± 0.43, *p* = 0.008), and tPRK (0.78 ± 0.48, *p* = 0.001), and was smallest in tPRK Xtra (0.66 ± 0.47, *p* < 0.001), Figure 4D; Table 4. Similarly, after correction for △CCT from pre to pos6m, preoperative bIOP and baseline DARatio2mm, the change in DARatio2mm between pre and pos6m in FS-LASIK was statistically higher than in tPRK (FS-LASIK vs. tPRK *p* < 0.001) and the corresponding Xtra group (FS-LASIK vs. FS-LASIK Xtra, *p* = 0.002), Table 4. Moreover, after these corrections, the changes in DARatio2mm in the FS-LASIK Xtra became statistically smaller than in the tPRK Xtra (*p* = 0.017). The changes of DARatio2mm indicate that FS-LASIK creates greater reductions in corneal stiffness, and benefits from greater biomechanical enhancement in subsequent CXL treatments, compared with tPRK, Figure 5D.

**FIGURE 6:**
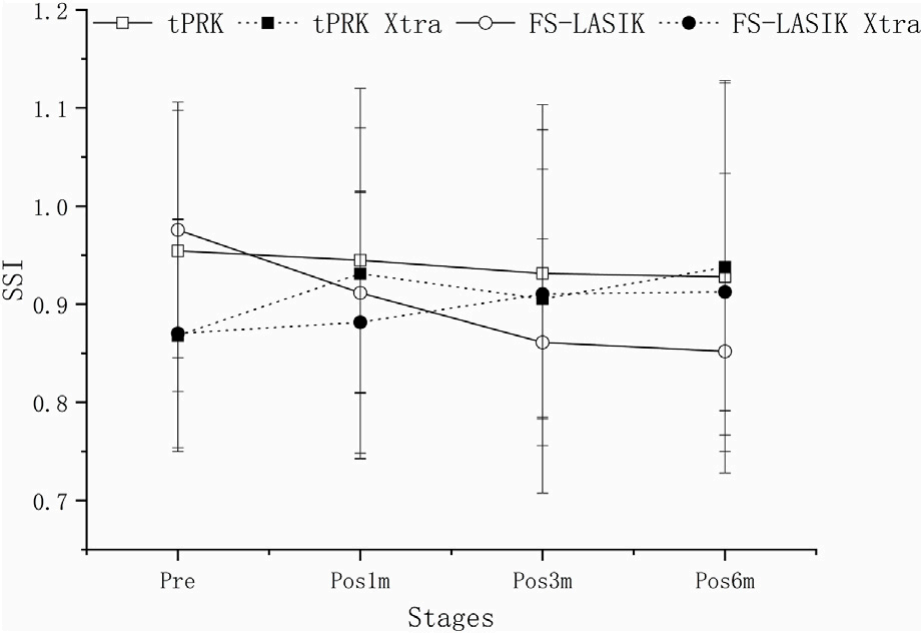
Change of SSI throughout all follow up stages in the four groups.

**FIGURE 7:**
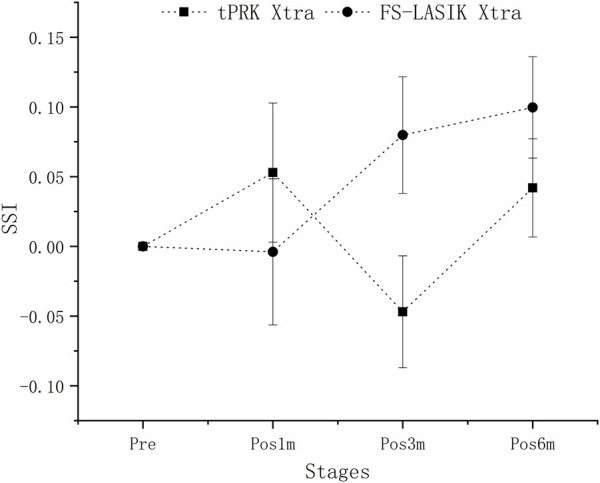
Isolating the effects of CXL: the estimated changes in SSI at all follow-up stages across the four groups. These changes are adjusted for preoperative bIOP, baseline biomechanical metrics, and △CCT from baseline to each follow-up stage.

SSI underwent significant decreases in the FS-LASIK group from pre to pos6m (*p* < 0.01), but not in the tPRK (*p* = 1.000), tPRK Xtra (*p* = 0.053) and FS-LASIK Xtra groups (*p* = 0.635), Figure 6. The decrease in SSI from pre to pos6m (ΔSSI) in FS-LASIK (-0.12 ± 0.10, -12.1 ± 7.9%) was statistically higher than in tPRK (-0.03 ± 0.22, or -1.5 ± 21.7%, *p* = 0.045), and was different from the two corresponding Xtra groups (all *p* < 0.01), Figure 6. ΔSSI also showed similar trends (*p* = 1.000) from pre to pos6m in the tPRK Xtra (0.07 ± 0.16, 8.4 ± 17.9%) and the FS-LASIK Xtra groups (0.04 ± 0.11, 5.6 ± 12.7%), while there were no significant differences of SSI in both groups. Meanwhile, the stiffening effect of CXL on SSI from pre to pos6m (ΔSSI) was statistically significant in FS-LASIK (FS-LASIK vs. FS-LASIK Xtra, *p* < 0.001) and in tPRK (tPRK vs. tPRK Xtra, *p* = 0.009). After correction for ASD, preoperative bIOP and baseline SSI, the change in SSI from pre to pos6m in FS-LASIK remained significantly higher than in tPRK (tPRK vs. FS-LASIK, *p* = 0.008), which indicates a greater reduction in stiffness from the loss of tension in the flap with FS-LASIK. The change in SSI was also higher than in FS-LASIK Xtra (FS-LASIK Xtra vs. FS-LASIK, *p* = 0.007). This indicates a greater reduction in stiffness without CXL in FS-LASIK and thus a positive stiffening effect of CXL. However, ΔSSI from pre to pos6m between tPRK and the corresponding Xtra group was non-significant after correction (tPRK vs. tPRK Xtra, *p* = 0.236), Table 4. ΔSSI results indicate that the stiffening effect of CXL may be less in tPRK than in FS-LASIK, as shown in Figure 7.

## 4 Discussion

The main objective of this study was to evaluate the corneal biomechanical responses to two of the most commonly used LVC procedures; tPRK and FS-LASIK and their cross-linked variations; tPRK Xtra and FS-LASIK Xtra. The effect of CXL in reducing corneal stiffness deterioration was higher in FS-LASIK than in tPRK which seems to compensate, to some extent, for the larger effect of FS-LASIK on stiffness reduction compared with tPRK.

However, while CXL caused significantly less stiffness reduction after both tPRK-Xtra and FS-LASIK-Xtra which was evident throughout the follow-up period (all *p* < 0.01), the stiffness increases remained much lower than the stiffness losses caused by the LVC procedures (all *p* < 0.01). Despite this large difference in effect, prophylactic CXL remains a useful technique, to counter some of the deleterious biomechanical effects of LVC surgeries and may be effective to reduce the risk of iatrogenic ectasia. This general outcome is compatible with the findings of earlier studies on the subject, and with the growing popularity of Xtra procedures (Kanellopoulos and Asimellis, 2015; Lee et al., 2017a).

Our study relied on five deformation parameters provided by the Corvis ST, three of which correlate with overall corneal stiffness, namely SP-A1, IIR and DARatio2mm, while the SSI, was designed to represent the tissue’s material stiffness. In all deformation parameters, there were indications of significant stiffness reductions at the first follow-up point after surgery (pos1m). SP-A1 experienced significant decreases of 27.8 ± 12.7% and 26.4 ± 21.1%, respectively with tPRK and FS-LASIK. The corresponding mean increases in IIR were 22.7 ± 14.4% and 31.5 ± 15.1%, in DA were 5.9 ± 9.4% and 8.9 ± 8.5%, and in DARatio2mm were 20.9 ± 10.8% and 29.6 ± 11.0%. Meanwhile, there were smaller stiffness reductions in Xtra groups compared with the corresponding non-Xtra groups. SP-A1 decreased by 23.1 ± 17.1% and 28.3 ± 11.2%, respectively with tPRK Xtra and FS-LASIK Xtra. There were also corresponding reductions of 15.5 ± 14.0% and 24.2 ± 12.7% in IIR, 1.3 ± 10.7% and 9.2 ± 10.4% in DA, and 10.9 ± 12.4% and 22.0 ± 10.6% in DARatio2mm in tPRK Xtra and FS-LASIK Xtra groups, respectively. Following pos1m and to the end of the follow-up period at pos6m, the differences in these parameters were, in most cases, small and non-significant except for significant increases of DA in tPRK, tPRK Xtra and FS-LASIK Xtra groups and significant increase of IIR in tPRK group.

These trends were generally in agreement with earlier reports except for a few minor differences. The postoperative decrease in SP-A1 in both tPRK and tPRK Xtra groups was similar to the results of a study by Lee et al. (2017a). In another study by Xu et al. (2017), the stiffness increases (indicated by decreases in highest concavity peak distance (PD) and DA) associated with using CXL after LASIK were not significant at all follow-up time points, but this may have been due to the small sample size employed. Further, the lower stiffness reductions noted in our study caused by CXL after both LASIK and tPRK surgeries were similar to the trends observed in two earlier studies, by Lee et al.(2017a, 2017b), Li et al. (2021). However, the non-significant results in DARatio2mm and SP-A1 noted in our study between the tPRK group and its corresponding Xtra group varied from those reported in these two earlier studies (Lee et al., 2017a; Li et al., 2021), which may be due to different crosslinking doses and eyes with risk for developing ectasia after LVC included in our study. These result were similar as our previous animal study (Bao et al., 2021). In the present study, total CXL energy doses varied from 2.1 J/cm^2^ to 2.7 J/cm^2^, according to the sum of risk scores, whereas both Lee et al. and Li et al. adopted a fixed total dose of 2.7 J/cm^2^ which is higher than us. Meanwhile, the previous study by Lee et al. (2017b) involved healthy myopia patients in both the tPRK and tPRK Xtra groups, whereas our study considered patients with high-risk postoperative ectasia for membership of the Xtra groups. This difference in group formation may have contributed to the differences in some of the biomechanical metrics considered in our and earlier studies.

Distinct from the overall stiffness represented by the other four Corvis parameters, the SSI parameter was designed to represent the cornea’s material stiffness independent of IOP and corneal geometry (Eliasy et al., 2019). The results showed significant improvement in ΔSSI in FS-LASIK Xtra group over FS-LASIK group, which is compatible with the large body of evidence depicting corneal stiffness increases with CXL (Kanellopoulos et al., 2015; Kling et al., 2017; Torres-Netto et al., 2020). However, notably, SSI was developed using finite element analysis in which tissue separation was not simulated (Eliasy et al., 2019). There are two separate regions in FS-LASIK and FS-LASIK Xtra with different biomechanical environments, the flap without biomechanical contribution and the residual stromal bed, which was not captured in the development of SSI. Thus, the reduction in SSI of FS-LASIK does not fully represent the material properties deterioration. Non-etheless, by the similar flap and RSB thickness adopted, it is still meaningful to compare the SSI changes from pre to pos6m between FS-LASIK and FS-LASIK Xtra. Although there were also different trends observed in two t-PRK groups, the changes were not significant. The SSI changes were also consistent with our previous animal experiments, which showed material stiffness increases caused by CXL after tPRK (Bao et al., 2021). In that study, the increases in SSI between pre-and post-Xtra surgery, were only significant in the tPRK Xtra group with a total CXL energy dose of 2.7 J/cm^2^ but not 1.8 J/cm^2^ (Bao et al., 2021).

Since the FS-LASIK flap made nearly no biomechanical contribution to the cornea, this procedure led to a smaller effective stromal thickness, and caused a larger reduction in corneal stiffness compared with the surface ablation procedure tPRK (Hashemi et al., 2017; Guo et al., 2019). If the flap is to be ignored biomechanically, the cornea would end up with a smaller effective stromal thickness after FS-LASIK than after tPRK. This effect is exaggerated by the differences in microstructure between the anterior and posterior parts of the stroma. With the anterior stroma having higher lamellae packing density, more interlacing, and being less hydrated and less easily swollen (Radner et al., 1998; Müller et al., 2001; Bergmanson et al., 2005; Meek and Knupp, 2015; Torres-Netto et al., 2020), the anterior stroma is known to be stiffer than the posterior tissue (Kanellopoulos and Asimellis, 2015). In other words, compared to tPRK, the residual stromal bed in FS-LASIK is thinner and composed of a higher proportion of the softer posterior stroma. Therefore, the combined effect of tissue separation and higher stiffness in the ablated anterior stroma leads to higher stiffness losses caused by FS-LASIK than by tPRK.

Although the results showed a trend for more biomechanical metrics such as DA, DARatio 2 mm and SSI which were significantly different between FS-LASIK Xtra and its corresponding non-Xtra group compared with tPRK Xtra, no statistical difference in these metrics was observed between the two Xtra groups from pre to pos6m. However, it is important to note that DARatio2mm did show a significant statistical difference between tPRK Xtra and FS-LASIK Xtra after correcting for covariates. Meanwhile, FS-LASIK showed greater weakening than tPRK without CXL according to DARatio2mm and SSI. Our results indicate that the stiffening effect after CXL dominates the biomechanical differences between the refractive surgery techniques with a greater impact on the LVC procedure with the greatest weakening. This result was consistent with a previous study, which indicated that the biomechanical weakening of different LVC retreatment options after SMILE seems to be small compared with the enhancement effect of accelerated CXL (9 mW/cm^2^, 10 min) (Kling et al., 2017).

If, on the other hand, CXL were applied after re-placing the flap, the expected outcome might have been the opposite. In that case, a large part of the cross-linking would have been consumed in stiffening the flap, leading to a potential risk of flap shrinking and contributing little to post-surgery corneal stiffness (Kanellopoulos and Asimellis, 2015). However, with CXL applied before re-placing the LASIK flap in our study, the fact that FS-LASIK had a smaller residual stromal thickness than tPRK meant that applying the Xtra treatment after the former surgery would stiffen a larger percentage of the posterior stromal thickness (lower PDLD and similar DLA) and lead to more significant biomechanical metric results of CXL. The difference in microstructure of anterior and posterior stroma may induce different stiffening effects in both parts, the increase ratio in posterior part is higher than anterior part after CXL consistent with previous studies (Lombardo et al., 2014; Beshtawi et al., 2016). Several biomechanical metrics were only pronounced in FS-LASIK (demonstrated by DA, DARatio 2 mm and SSI) as explained above. LASIK flap creation significantly reduced stiffness in the anterior stroma (Hashemi et al., 2017; Guo et al., 2019), while CXL may only take effect in the residual stroma under the flap.

Steps were taken to ensure patient safety in FS-LASIK Xtra group, especially with the direct irradiation of the RSB. A higher intensity (30 mW/cm^2^) transfer compared to standard CXL protocol was adopted, and the residual stromal thickness was never below 320 µm. As a result of this precaution, the demarcation line was shallower compared with those reported with the Dresden protocol (Aldahlawi et al., 2015; Aldahlawi et al., 2016). All the PDLD (232.4 ± 50.3 μm, range 128–314 μm) in FS-LASIK Xtra were higher than 70 μm, which was considered the safety threshold in a recent study by Hafezi et al. (2021). Further, the endothelial cell count in FS-LASIK Xtra remained similar (*p* > 0.05) before (2,857 ± 344 cells/mm^2^) and after (2,742 ± 296 cells/mm^2^) surgery. Meanwhile, the safety index was greater than one in the FS-LASIK Xtra group, which also indicates the operation was safe. Ultimately, none of the patients developed serious complications within 6 months of follow-up. Thus, based on the above results, this FS-LASIK Xtra positively affects biomechanical outcomes without endothelial damage or visual acuity threat.

The major limitation of this investigation was that it was a non-randomized study with different baselines between groups. This was addressed by using baseline parameters as co-variates in the statistical analysis. In addition, there were multiple CXL procedures in the XTra groups. Also, the study was limited to data obtained over a follow-up period of 6 months and thus it does not extend to late ectasia. A further study with a larger sample size and longer follow-up would be important to investigate the biomechanical enhancement efficacy of CXL in Xtra surgeries.

In summary, we evaluated the effectiveness of CXL in compensating for the stiffness losses caused in the cornea by two forms of LVC, namely tPRK and FS-LASIK. However, three CXL procedures (2.1, 2.4 and 2.7 J/cm^2^) were not sufficient to fully address the reductions caused by the LVC procedures. The biomechanical enhancement of CXL was higher in LASIK than in tPRK, and that phenomenon was useful considering the greater effect of FS-LASIK on corneal biomechanics losses than tPRK. Based on our results, the combined application of CXL with tPRK and FS-LASIK would have a positive effect in reducing the losses in corneal biomechanics and may reduce the risk of developing iatrogenic ectasia.

## Data Availability

The raw data supporting the conclusion of this article will be made available by the authors, without undue reservation.
