# Supplementary material for: Effect of corneal cross-linking on biomechanical changes following transepithelial photorefractive keratectomy and femtosecond laser-assisted LASIK
Source: Front Bioeng Biotechnol. 2024 Mar 15;12:1323612. doi: 10.3389/fbioe.2024.1323612 (PMC10978754; doi:10.3389/fbioe.2024.1323612)
Supplement: Supplementary file 1 [file Table1.docx]

Supplementary Material

Effect of Cross-linking on Corneal Stiffness Changes following Transepithelial Photorefractive Keratectomy and Femtosecond laser-assisted LASIK

Wen Chen^†^, FangJun Bao^†^, Cynthia J Roberts, Jia Zhang, XueFei Li, JunJie Wang, Anas Ziad Masoud Abu Said, Kevin Nguelemo Mayopa, YaNi Chen, XiaoBo Zheng, Ashkan Eliasy, Ahmed Elsheikh^*^, ShiHao Chen^*^

*** Correspondence:**Ahmed Elsheikh
Ahmed.Elsheikh@liverpool.ac.uk

ShiHao Chen
[chenle@rocketmail.com](mailto:chenle@rocketmail.com)

**Supplement Table 1**. Relationship between basic biometric parameters and biomechanical terms.

| Parameter | Increased Resistance to Deformation | Increased Stiffness | Increased Material Stiffness | Biomechanical enhancement |
| --- | --- | --- | --- | --- |
| SP-A1(mmHg/mm) | Increased | Increased | Not applicable | Increased |
| IIR (mm) | Decreased | Decreased | Not applicable | Decreased |
| DA (mm) | Decreased | Decreased | Not applicable | Decreased |
| DARatio2mm | Decreased | Decreased | Not applicable | Decreased |
| SSI | Not applicable | Not applicable | Increased | Increased |

Stiffness denotes the cornea's resistance to deformation, also known as geometric stiffness. Material stiffness is defined by the corneal material rigidity, commonly described by the tangent modulus. Biomechanical enhancement' encompasses improvements in both stiffness and biomechanical stiffness, indicating a comprehensive increase in the cornea's ability to resist mechanical stress.
