# Supplementary material for: Effect of corneal cross-linking on biomechanical changes following transepithelial photorefractive keratectomy and femtosecond laser-assisted LASIK
Source: Front Bioeng Biotechnol. 2024 Mar 15;12:1323612. doi: 10.3389/fbioe.2024.1323612 (PMC10978754; doi:10.3389/fbioe.2024.1323612)
Supplement: Supplementary file 2 [file Table2.docx]

Supplementary Material

Effect of Cross-linking on Corneal Stiffness Changes following Transepithelial Photorefractive Keratectomy and Femtosecond laser-assisted LASIK

Wen Chen^†^, FangJun Bao^†^, Cynthia J Roberts, Jia Zhang, XueFei Li, JunJie Wang, Anas Ziad Masoud Abu Said, Kevin Nguelemo Mayopa, YaNi Chen, XiaoBo Zheng, Ashkan Eliasy, Ahmed Elsheikh^*^, ShiHao Chen^*^

*** Correspondence:**Ahmed Elsheikh
Ahmed.Elsheikh@liverpool.ac.uk

ShiHao Chen
[chenle@rocketmail.com](mailto:chenle@rocketmail.com)

**Supplement Table 2** Significance of changes in the corneal biomechanical metrics between pre and pos6m in the four surgery groups.

|  | p values | SP-A1(mmHg/mm) | IIR(mm) | DA(mm) | DA Ratio2mm | SSI |
| --- | --- | --- | --- | --- | --- | --- |
| tPRK | p (pre vs pos1m) | <0.001** | <0.001** | 0.004** | <0.001** | 0.735 |
|  | p (pre vs pos3m) | <0.001** | <0.001** | <0.001** | <0.001** | 1.000 |
|  | p (pre vs pos6m) | <0.001** | <0.001** | <0.001** | <0.001** | 1.000 |
|  | p (pos1m vs pos3m) | 1.000 | 0.655 | 1.000 | 0.223 | 1.000 |
|  | p (pos1m vs pos6m) | 1.000 | 0.033* | 0.005** | 0.246 | 0.094 |
|  | p (pos3m vs pos6m) | 1.000 | 1.000 | 0.200 | 1.000 | 0.582 |
| tPRK Xtra | p (pre vs pos1m) | <0.001** | <0.001** | 1.000 | <0.001** | 0.001** |
|  | p (pre vs pos3m) | <0.001** | <0.001** | <0.001** | <0.001** | 1.000 |
|  | p (pre vs pos6m) | <0.001** | <0.001** | <0.001** | <0.001** | 0.053 |
|  | p (pos1m vs pos3m) | 0.515 | 0.037* | 0.014* | 0.051 | 0.103 |
|  | p (pos1m vs pos6m) | 1.000 | 1.000 | 0.001** | 0.166 | 0.266 |
|  | p (pos3m vs pos6m) | 1.000 | 0.234 | 1.000 | 1.000 | 1.000 |
| FS-LASIK | p (pre vs pos1m) | <0.001** | <0.001** | <0.001** | <0.001** | 1.000 |
|  | p (pre vs pos3m) | <0.001** | <0.001** | <0.001** | <0.001** | 0.002** |
|  | p (pre vs pos6m) | <0.001** | <0.001** | <0.001** | <0.001** | <0.001** |
|  | p (pos1m vs pos3m) | 1.000 | 1.000 | 0.924 | 1.000 | 0.336 |
|  | p (pos1m vs pos6m) | 0.737 | 1.000 | 0.005** | 1.000 | 0.044* |
|  | p (pos3m vs pos6m) | 1.000 | 1.000 | 0.579 | 1.000 | 1.000 |
| FS-LASIK Xtra | p (pre vs pos1m) | <0.001** | <0.001** | <0.001** | <0.001** | 1.000 |
|  | p (pre vs pos3m) | <0.001** | <0.001** | 0.001** | <0.001** | 1.000 |
|  | p (pre vs pos6m) | <0.001** | <0.001** | <0.001** | <0.001** | 0.635 |
|  | p (pos1m vs pos3m) | 1.000 | 0.524 | 1.000 | 0.693 | 1.000 |
|  | p (pos1m vs pos6m) | 1.000 | 0.059 | 0.482 | 1.000 | 1.000 |
|  | p (pos3m vs pos6m) | 1.000 | 1.000 | 1.000 | 1.000 | 1.000 |

SP-A1 means the stiffness parameter at first applanation, IIR means integrated inverse radius, DA means the deformation amplitude at the apex, DARatio2mm means the ratio of deformation amplitude between the apex and 2mm from the apex, SSI means the stress-strain index provided by Corvis ST, * Means p <0.05, ** means p <0.01.
