# Supplementary material for: Effect of corneal cross-linking on biomechanical changes following transepithelial photorefractive keratectomy and femtosecond laser-assisted LASIK
Source: Front Bioeng Biotechnol. 2024 Mar 15;12:1323612. doi: 10.3389/fbioe.2024.1323612 (PMC10978754; doi:10.3389/fbioe.2024.1323612)
Supplement: Supplementary file 3 [file Table3.docx]

Supplementary Material

Effect of Cross-linking on Corneal Stiffness Changes following Transepithelial Photorefractive Keratectomy and Femtosecond laser-assisted LASIK

Wen Chen^†^, FangJun Bao^†^, Cynthia J Roberts, Jia Zhang, XueFei Li, JunJie Wang, Anas Ziad Masoud Abu Said, Kevin Nguelemo Mayopa, YaNi Chen, XiaoBo Zheng, Ashkan Eliasy, Ahmed Elsheikh^*^, ShiHao Chen^*^

*** Correspondence:**Ahmed Elsheikh
Ahmed.Elsheikh@liverpool.ac.uk

ShiHao Chen
[chenle@rocketmail.com](mailto:chenle@rocketmail.com)

|  | Stages | tPRK | tPRK Xtra | FS-LASIK | FS-LASIK Xtra |
| --- | --- | --- | --- | --- | --- |
|  |  | Means ± SD | | | |
| Corneal Densitometry (GSU) | Pre | 12.79±2.14 | 14.44±1.98 | 11.58±1.09 | 12.29±2.04 |
|  | Pos1m | 12.28±1.36 | 15.46±1.82 | 10.98±1.30 | 12.67±2.32 |
|  | Pos3m | 11.49±1.41 | 13.35±2.06 | 10.43±1.44 | 12.14±1.97 |
|  | Pos6m | 11.81±2.41 | 12.48±1.38 | 10.23±1.23 | 12.94±3.18 |

**Supplement Table 3** Change in corneal densitometry over the total area after different surgeries of the four surgery groups.

GSU means gray scale units.
