# Supplementary material for: Effect of corneal cross-linking on biomechanical changes following transepithelial photorefractive keratectomy and femtosecond laser-assisted LASIK
Source: Front Bioeng Biotechnol. 2024 Mar 15;12:1323612. doi: 10.3389/fbioe.2024.1323612 (PMC10978754; doi:10.3389/fbioe.2024.1323612)
Supplement: Supplementary file 4 [file Table4.docx]

Supplementary Material

Effect of Cross-linking on Corneal Stiffness Changes following Transepithelial Photorefractive Keratectomy and Femtosecond laser-assisted LASIK

Wen Chen^†^, FangJun Bao^†^, Cynthia J Roberts, Jia Zhang, XueFei Li, JunJie Wang, Anas Ziad Masoud Abu Said, Kevin Nguelemo Mayopa, YaNi Chen, XiaoBo Zheng, Ashkan Eliasy, Ahmed Elsheikh^*^, ShiHao Chen^*^

*** Correspondence:**Ahmed Elsheikh
Ahmed.Elsheikh@liverpool.ac.uk

ShiHao Chen
[chenle@rocketmail.com](mailto:chenle@rocketmail.com)

**Supplement Table 4** Significance of changes in the CCT and the mean corneal densitometry over the total area between pre and pos6m in the four surgery groups.

|  | p values | CCT | Corneal Densitometry (GSU) |
| --- | --- | --- | --- |
| tPRK | p (pre vs pos1m) | <0.001** | 0.674 |
|  | p (pre vs pos3m) | <0.001** | 0.004** |
|  | p (pre vs pos6m) | <0.001** | 0.218 |
|  | p (pos1m vs pos3m) | 1.000 | 0.041* |
|  | p (pos1m vs pos6m) | 0.111 | 1.000 |
|  | p (pos3m vs pos6m) | 0.391 | 1.000 |
| tPRK Xtra | p (pre vs pos1m) | <0.001** | 0.015* |
|  | p (pre vs pos3m) | <0.001** | 0.034* |
|  | p (pre vs pos6m) | <0.001** | <0.001** |
|  | p (pos1m vs pos3m) | <0.001** | <0.001** |
|  | p (pos1m vs pos6m) | <0.001** | <0.001** |
|  | p (pos3m vs pos6m) | 0.003** | 0.221 |
| FS-LASIK | p (pre vs pos1m) | <0.001** | 0.391 |
|  | p (pre vs pos3m) | <0.001** | 0.015* |
|  | p (pre vs pos6m) | <0.001** | 0.026* |
|  | p (pos1m vs pos3m) | 0.018* | 0.340 |
|  | p (pos1m vs pos6m) | 0.267 | 0.668 |
|  | p (pos3m vs pos6m) | 1.000 | 1.000 |
| FS-LASIK Xtra | p (pre vs pos1m) | <0.001** | 1.000 |
|  | p (pre vs pos3m) | <0.001** | 1.000 |
|  | p (pre vs pos6m) | <0.001** | 1.000 |
|  | p (pos1m vs pos3m) | 1.000 | 0.487 |
|  | p (pos1m vs pos6m) | 0.059 | 1.000 |
|  | p (pos3m vs pos6m) | 0.971 | 0.358 |

GSU means gray scale units.
